# Exploiting Bi-Directional Self-Organizing Tendencies in Team Sports: The Role of the Game Model and Tactical Principles of Play

**DOI:** 10.3389/fpsyg.2019.02213

**Published:** 2019-10-09

**Authors:** João Ribeiro, Keith Davids, Duarte Araújo, José Guilherme, Pedro Silva, Júlio Garganta

**Affiliations:** ^1^CIFI2D, Centre of Research, Education, Innovation and Intervention in Sport, Faculdade de Desporto, Universidade do Porto, Porto, Portugal; ^2^Centre for Sports Engineering Research, Sheffield Hallam University, Sheffield, United Kingdom; ^3^CIPER, Faculdade de Motricidade Humana, Universidade de Lisboa, Lisbon, Portugal

**Keywords:** self-organization tendencies, bi-directional dynamical processes, game model, tactical principles of play, synergy formation, team sports

## Abstract

Research has revealed how inherent self-organizing tendencies in athletes and sports teams can be exploited to facilitate emergence of dynamical patterns in synergy formation in sports teams. Here, we discuss how game models, and associated tactical principles of play, may be implemented to constrain co-existing *global-to-local* and *local-to-global* self-organization tendencies in team sports players during training and performance. Understanding how to harness the continuous interplay between these co-existing, bi-directional, and coordination tendencies is key to shaping system behaviors in sports training. Training programs are traditionally dominated by designs, which shape the self-organizing tendencies of players and teams at a *global-to-local* scale by coaches imposing a tactical/strategical plan with associated tactical principles of play. Nevertheless, recent research suggests that performers also need to be provided with opportunities to explore self-organizing tendencies that emerge at the *local-to-global* scale in training. This directional tendency in synergy formation can be facilitated by players being given opportunities to actively explore different adaptive and innovative performance solutions, coherent with principles of play circumscribed in an overarching game model. Developing methods (coaching sessions rooted on principles of dynamical systems theory that foment the development of such local-to-global relations) to exploit the continuous interplay between these co-existing tendencies within sports teams may promote more effective and efficient athlete skill training programs, in addition to enhancing performance.

## Introduction

In past decades, there has been a concerted attempt to conceptualize sports teams as complex adaptive systems (CAS) (Davids et al., 2005; Araújo and Davids, 2016). Such systems display similar tendencies (e.g., self-organization under constraints, pattern forming dynamics, synergy formation, emergent behaviors, and sensitive dependence on initial conditions) like those observed in other collective systems in nature (e.g., ant colonies, schools of fish, flocks of birds, and human communities) (Duarte et al., 2012; Riley et al., 2012). In such systems, emergent behaviors and self-organization under constraints appear to be instrumental in explaining how collective-system behaviors emerge. For example, in team sports, synergetic relations arise from the continuous individual component interactions (i.e., between players) (Vilar et al., 2012).

On the one hand, system order emerges from the self-organizing interactions of individual components without ordering from an external agent (i.e., an executive, coach or commander) (Halley and Winkler, 2008). On the other hand, emergence typically depicts a (global) system property (e.g., tactical patterns of behaviors in a sports team) that is not reducible to properties of individual system components (e.g., local interactions of individual players), from which it arises (Halley and Winkler, 2008). Both levels of interaction are intrinsically connected, giving rise to systems displaying both local-to-global and global-to-local self-organization tendencies (Riley et al., 2012). Local-to-global self-organizing processes imply that rich patterns of behavior observed in a complex adaptive system is constrained by local interactions generated through cooperative or interactive behaviors among system components (e.g., fish in a school, a murmuration of flocking birds or team players in sport) (Riley et al., 2011). Conversely, global-to-local self-organizing processes imply that global system behaviors govern or constrain local interactions of individual system components in a top-down fashion, exhibiting circular causality (Kelso, 1995; Araújo and Davids, 2016). Such circular causality processes evidenced in both global-to-local and local-to-global self-organizing processes signify, for example, that players may interact locally with their nearest teammates (e.g., retrieving the ball from the pressure zone after recovering it) to produce more complex set of behaviors. Such set or behaviors are expressed at higher levels, with players behaving as a collective social unit (e.g., increases in team width and length through increases of players’ interpersonal distance values after recovering the ball). After the attainment of considerable levels of team width and length, there are some players that may need to cover crucial regions of the field in case of a momentarily ball-possession lose.

Essentially, circular causality (also known as non-linear causality) can be differentiated from linear causality due to the causal relationships established between the whole system and its constituents. While models involving linear causality follow a single, linear direction (event A causes an effect in B, while B has no demonstrable effect on A), non-linear causality depicts a circular causation between the whole system and its parts, involving global and local (bi-directional) self-organizing tendencies. Thus, linear causality in systems is characterized by processes of unidirectional flow of causation between both micro and macro levels, where higher levels (global system dynamics) are the unique result of the interactions between the constituent parts of the system (Raia, 2008). However, in non-linear systems with circular causality (e.g., sports teams), there is potential for a bi-directional flow to causation. This property of CAS reflects the constant interplay between both global-to-local and local-to-global dynamics in shaping system behaviors. For example, the article by Duarte et al. (2012) enlighted how interactions among grouping individuals (i.e., the players) scale to global collective system behaviors. These investigators proposed that teams can be viewed as functional integrated superorganisms, despite also revealing functional specialization, i.e., interindividual variation derived from genetic heritage, previous experiences, etc. Teams conceptualized as superorganisms reveal highly coordinated patterns in which the actions of individual players constrain and are constrained by the actions of neighboring players (teammates and/or opponents) toward the mutually exclusive goals of the collective. Importantly, research has provided substantial evidence that locally created information (e.g., approaching velocities of teammates and opponents, interpersonal distances) allows players to co-regulate actions with others (teammates and/or opponents) and the environment. Such process allows players to self-organize behaviors into newly formed structures, expressed at a global scale. These insights have some important implications when extrapolated to sports performance contexts. They signify, for instance, that individual players in sports teams co-adapt their actions to form specific intended collective structures (a systematic whole such as a tactical pattern of play). These system patterns emerge from players’ local interactions (local-to-global effects) under the dynamic constraints of competitive performance environments (Passos et al., 2013). In turn, collective organizational structures can shape the dynamical, interpersonal interactions of individual players competing and cooperating with each other in a top-down process (global-to-local effects). Such processes demonstrate the deeply entwined interconnections of bottom-up (local-to-global) and top-down (global-to-local) processes in the continuous (re)organization of CAS.

Here, we outline an argument that traditional coaching methods may be failing to exploit the bi-directional influence of dynamics in sports teams as CAS by over-valuing the influence of *global-to-local* tendencies in formation of synergetic relations between players in training (Araújo and Davids, 2009). A major example of overarching task constraints that strongly influence the global-to-local self-organizing tendencies of players and teams during competitive team sports performance is the game model. This is the overarching, planned, tactical/strategical approach adopted, and tactical principles of play, conceived by coaches to enhance player functionality in specific sub-phases of play (Garganta, 1997; Guilherme, 2004). There is clear relevance of a game model and tactical principles of play in shaping individual and collective self-organizing behaviors of players and teams. However, there has been little effort directed to understanding how *local-to-global* tendencies for self-organization can be harnessed in producing adaptive teams, which can rapidly and effectively adjust and diversify tactical patterns of behavior as competitive performance conditions change (please see Passos et al., 2013). Successful team sports performance requires a complex and intertwined relationship between co-existing global-to-local and local-to-global self-organizing tendencies in optimizing team functionality. The bi-directional tendency of synergy formation between team sports athletes is a performance aspect that has been completely disregarded by the sports sciences community.

This void in the literature may be due to a lack of understanding of how a game model and tactical principles of play may impact as potent constraints in shaping self-organization tendencies within sports teams. Therefore, this paper aims to clarify how a game model, and associated tactical principles of play, could constrain both global-to-local and local-to-global self-organization tendencies within sports teams. Furthermore, we discuss how coaches could contribute to enhancing the impact of both self-organizing tendencies in the design of training programs to enhance synergy formation between cooperating teammates.

## Defining a Game Model and Tactical Principles of Play

Previous literature (e.g., Bompa, 1994; Castelo, 1994; Guilherme, 2004) has emphasized that a game model provides a framework to map a set of characteristics to bound the implementation of defensive/offensive principles or systems of play in team sport athletes (including player roles and tactical system during play). Essentially, a game model encompasses tactical patterns of play, considered of fundamental importance for system (team) organization and functioning. A tactical principle of play encompasses a set of basic game rules (previously defined by the team coaches) that constrain the actions of the players and the teams toward intended performance outcomes for specific sub-phases of play (Garganta, 1997). Such sub-phases of play can be divided into the attacking phase (i.e., when team A is in possession of the ball – offensive organization) and the defending phase (when team B does not have the ball – defensive organization). Moreover, when team A loses ball possession (attack–defense transition or defensive transition), they try incessantly to recover it again, while team B gains ball possession (defense–attack transition or attacking transition) and seek to create goal-scoring opportunities.

Additionally, these principles articulate among themselves and represent a basic “level of process goal setting” that shapes the cooperative interactions and behaviors of players, individually and collectively, helping them to seek tactical solutions for contingencies emerging during competition (Garganta and Pinto, 1994; Garganta, 1997). Importantly, the game model itself appears to emerge not from a top-down process, but rather through a bottom-up process, since it is dependent on several interacting constraints such as the coaches’ ideas, capacities of the available players, the “form of life” of the club (Rothwell et al., 2018), the coaching and sport science support staff, and the financial constraints of the available budget.

On the other hand, the term “tactic” has its origins in the middle of the 17th century and was originally used in military activities. According to Gelfand and Tsetlin (1962), tactics can be understood as the skill of employing available means to accomplish an end. Traditionally, this concept has long been utilized by team sports coaches to refer to specific patterns of play used during competition to achieve a specific objective (e.g., overcome opposition defensive organization, avoid defeat, achieve a specific outcome like negate opposition strength in attack or exploit a weakness). On the other hand, team strategy is related to specific tactics and can be viewed as a more general, long-term, and overarching concept highlighting how teams exploit the use of their resources (defensive strategy or attacking strategy) (Garganta, 1997; Davids et al., 2005). Strategies can be based, for example, on detailed gathering and sharing of knowledge about different patterns of play (e.g., exploring the opponent weaknesses when defending). Hence, tactics is expressed through a set of observable behaviors or tactical patterns of play aimed to achieve a specific end, which in turn are constrained by a defined strategy. A key distinction here is that tactical patterns of play can sometimes emerge from local to global interactions as adaptive performance behaviors can alter quickly as opponent adapt their formations or tactics (information required for teams’ organization and functioning is constantly changing) (Duarte et al., 2012; Passos et al., 2013; Silva et al., 2013). In contrast, strategies constitute more long-term processes that involved experimentation, implementation, and formulation, which are constrained by global-to-local interactions (Silva et al., 2013). Here, we contend that tactics and strategy work jointly as a means to an end. Both comprehend intertwined concepts such as experimentation, implementation, and formulation for enhancing team performance. For instance, if a given principle advocated by a team coach is to progress with the ball upfield through both left- and right-hand sides of the opposing team, the players need to tactically adopt certain behaviors that favor team objectives in order to attract the opponents toward the midfield regions in order to create available space to be explored.

Principles of play articulate with and influence a team’s game model as evidenced at different levels of team organization (macro/global to micro/local, and vice versa) (Garganta, 1997; Guilherme, 2004). This relationship shapes how performance strategies can be elaborated and implemented in competition. Such a body of knowledge can be used for developing specialized player development programs, as well as for the establishment of theoretical and practical frameworks for periodizing team training and performance in different sub-phases of play (Seifert et al., 2018). Through careful manipulation of constraints, coaches can help team players to perceive shared affordances that support desired team behaviors (in collective principles of play) through practice. Such affordances are available and attainable only by groups of individuals during specialized cooperative actions, presenting possibilities for group actions and do not exist outside this cooperative dynamic environment. This is particularly important because the perception of an individual affordance by an individual player, cooperating with others in a group, is dependent on the perception of *affordances of* and *for* others and of his/her own affordances by others (Silva et al., 2013).

For example, in football, a midfielder with the ball perceives an opportunity to make a long pass to a winger, who initiates a penetrating run behind the defensive line, through anticipating this affordance for his/her teammate. A third player, the striker, can also time a run into the penalty box to receive an anticipated cross from the winger, because he also perceived the affordances for the two teammates, while, at the same time, providing them with another affordance – receiving a cross to score. This collective movement pattern may, therefore, constitute how a principle of play, penetration, is sustained by the coordination of individual affordances that are shared locally to form a collective affordance for an attacking sub-system of players.

## Exploiting Global-To-Local and Local-To-Global Self-Organizing Tendencies in Sports Teams

Essentially, global-to-local and local-to-global tendencies provide two main mechanisms through which self-organization gives rise to the emergence of a specific identity (Vernon et al., 2015), expressed by synergetic team behavioral patterns during performance.

The inter-level causality evidenced by both directional processes, in team sports, may function as a heterarchy rather than a hierarchy, meaning that there is not a dominant relation of the whole relative to the parts in the system self-organizing tendencies. Imagine a situation in which a team sport coach (e.g., basketball, handball, volleyball, football, etc.) defines a specific tactical principle for implementation in a particular sub-phase of play. Even though this principle acts at a global-to-local scale to constrain the individual dynamics of players at a local-to-global scale, players will always have the ability to use their unique resources (physical, perceptual–cognitive skills, etc.) to continuously reshape the proposed principle through development of local-to-global self-organizing tendencies. Hence, there is a reciprocal (but not symmetrical) interaction between both levels of self-organizing tendencies.

Thus, it is important to note that inter-level causal relations flow in both directions (global-to-local and local-to-global) continuously and mutually influencing each other. In team sports, a game model and tactical principles of play may be influential at a global-to-local scale, thus shaping the local interactions of players, which function at a local-to-global scale. For instance, in football (soccer), a coach may have defined as a tactical principle of play for the attacking sub-phase, to circulate the ball through the right- and left-hand sides of the opposing defense, aiming to cross the ball toward a targeted player. This principle (acting at a global-to-local scale) can influence the local-to-global self-organizing tendencies of players by leading them to attract the opponents toward the center of the pitch in order to create and explore possible empty spaces left in both right- and left-hand sides of the opposing team. Successful sports teams can harness these co-existing tendencies to enhance team synergy formation and performance, without solely relying on a game model framework imposed by a coach.

Global-to-local effects do not take the same form as local-to-global influences. The former typically entail modifications in order parameters (variables that capture a system’s global state emerging from the interaction of its components) (Kelso, 1995, 2012). In contrast, local-to-global tendencies are captured in changes in interacting dynamical variables (e.g., circumstantial numerical relations, acting upon passing opportunities, and co-positioning of players), which constrain how players coordinate and regulate actions, supporting effective communications with other teammates (Araújo and Davids, 2016). Coaches typically tend to exert influence at the global-to-local scale, by managing a strategy, suggesting certain tactical behaviors, and designing selected practice tasks (in a top-down process). These externally imposed influences can provide an overarching constraint, operating at a slower timescale on players’ perceptions, decisions, and actions as they seek to achieve specific performance outcomes. Importantly, the design of practice tasks is conditioned by specificities of the game model conceptualized by a coach, particularly with respect to strategical principles of play, which, altogether, interact globally-to-locally, to constrain individual and collective performance. Alternatively, team players’ continuous local interactions function at the local-to-global scale (changing at a faster timescale than the global-to-local scale) and can support the emergence of specific tactical patterns of behavior (in a bottom-up process) in resolving a performance issue during play (e.g., rapid re-organization to prevent an underload in one part of the playing area).

This type of self-organizing tendency is paramount in athlete development programs since local-to-global interactions are instrumental in developing the co-adaptive processes (see Passos et al., 2016) needed in team games players. Indeed, they are the basis of co-adaptation between players because the constraints acting on the spontaneous self-organizing tendencies are wide-ranging (Passos et al., 2016). Thus, they should be the dominant self-organizing tendencies that athletes should be exposed to at all stages of their careers, especially at younger ages (Passos et al., 2016). Indeed, the promotion of such tendencies during practice and competition is fundamental for developing “intelligent performers in sport,” capable of solving emerging performance and tactical (organizational) problems from experience. The development of learners in sport as “intelligent performers” and active decision makers has become a central aim in Physical Education curriculum documents worldwide. Across the world, recent government publications, national standards, professional bodies, and curriculum documents have recognized the role of thinking skills in physical education. In the United Kingdom, the National Curriculum Physical Education (NCPE), the NASPE (National Association for Sport and Physical Education) in the United States, and the Queensland Physical Education Senior Syllabus (Queensland Studies Authority, 2004) incorporate outcomes in all three of the major domains of learning: psychomotor, cognitive, and affective in their definition of a physically educated person (see Metzler, 2005; Byra, 2006; National Association for Sport and Physical Education USA, 2009; Queensland Studies Authority, 2010; Department for Education, 2013; Australian Curriculum, Assessment and Reporting Authority, 2015; Moy et al., 2019).

For example, the Queensland Studies Authority (2010, p. 3) states that “Intelligent performance is characterized by high levels of cognitive functioning, using both rational and creative thought. Students are decision makers engaged in the active construction of meaning through processing information related to their personal experience and to the study of physical activity.”

In sport, environmental, individual and task constraints can alter every time an action is performed, and an “intelligent performer” is viewed as a skilled, adaptive individual who can achieve task goals or individualized functional performance solutions in different ways (Davids et al., 2006; Araújo and Davids, 2011). Adaptive skilled behavior, rather than being imposed by a pre-existing plan, model, or structure, emerges from this confluence of constraints as athletes seek individualized solutions to a specific performance problem through active exploration of a learning environment. Continuous interactions with performance contexts in practice enable learners to seek and perceive information to regulate actions, enhancing their knowledge and understanding of a competitive environment. This process will be most effective if coaches develop flexible and adaptable performers (they can rapidly alter and/or adapt their behaviors to changing performance constraints) who can perceive information on opposition tactical patterns and respond quickly and effectively during play.

## Malleability of the Game Model and Tactical Principles of Play for Developing Effective Self-Organizing Tendencies in Team Sports

A challenging task for many coaches in team sports is to provide suitable learning environments for developing adaptive, “intelligent performers.” Intelligent performers in sport have cognitions, perceptions, and actions deeply intertwined in all phases of play (Davids et al., 2015). They know how to use information to regulate their actions and are autonomous problem solvers, without constantly resorting to a coach for performance solutions (Davids et al., 2015). A fundamental misconception regarding the role of a game model and tactical principles of play is to consider them as being achieved by *pre-planned* and *pre-established* actions or mechanized movements that players should faithfully rehearse during practice (e.g., in shadow play during training when a team perform patterns of play without opponents present). Such misconceptions may negatively influence the frameworks for athlete development programs for development of expertise and performance.

Thus, it is important to emphasize that the principles of play underlying a game model stipulated with a coach for each sub-phase of play should not be rigid and inflexible (Garganta, 1997; Guilherme, 2004). Rather, they must be flexible and open to the shared affordances players utilize from it (in terms of possibilities for action) and how athletes can be prepared to exploit affordances by enhancing their action readiness [see Frijda (1986, 2007) for detailed descriptions on this concept]. During practice, coaches should allow players to freely explore a wide variety of performance solutions when guided by a particular principle of play (Guilherme, 2004). This approach to practice would facilitate co-adaptation of players using local information sources (e.g., teammate and opponent displacements, or co-positioning in a playing area relative to a scoring target or area markings) (Passos et al., 2016). This practice approach would enable athletes to explore principles of play through exploiting shared affordances and synergies formed through local-to-global self-organizing tendencies (Davids et al., 2015; Silva et al., 2013, 2016). Indeed, previous research (e.g., Araújo et al., 2004; Silva et al., 2013; Davids et al., 2015) have stressed the importance of athletes to perceive affordances by learning to detect relevant information sources that support successful task performance. Moreover, experiencing a vast amount of tactical solutions for achieving a specific principle of play will lead to adaptive movement variability, which is key for developing expert performers (Davids et al., 2015).

**FIGURE 1:**
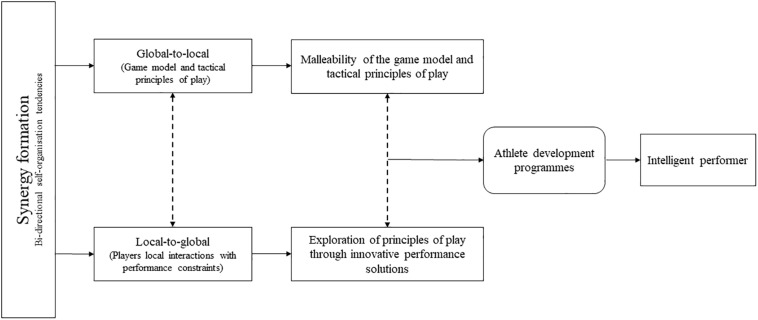
Developing synergy formation processes through exploitation of self-organization tendencies in a global-to-local and local-to-global direction.

It is important to ensure that athletes cannot only co-adapt to one major constraint in synergy formation: harnessing global-to-local self-organizing tendencies (i.e., the coach staff imposing and implementing a tactical plan). Rather, athletes need to be facilitated in exploiting available local-to-global synergy forming tendencies by co-adapting to a variety of interacting personal, task and environmental constraints during performance and practice. They can achieve these adaptive skills by exploring a range of performance solutions coherent with principles of play (see Figure 1).

A key take-home message of this article is that for a given principle of play, there exists a multitude of performance solutions or coordination patterns (synergies) attainable by performers. Edelman and Gally (2001) identified “degeneracy” as an inherent property of CAS signifying “the ability of elements that are structurally different to perform the same function or yield the same output” (p. 13, see also Seifert et al., 2016). This conceptualization provides a theoretical foundation to propose why performers in sport should be encouraged to explore solutions for achieving task goals aligned with principles of play in team sports. When framed as a methodological approach for sport practice, it supports athletes in combining their unique resources (e.g., physical, technical, tactical, perceptual skills and awareness, psychological attributes, and emotional control) to seek and explore tactical behaviors through team synergy formation.

## A Tactical Landscape for Developing Tactical Principles of Play Circumscribed in a Game Model

A fundamental question that can emerge from this opinion piece is: how can coaches develop through training both individual and collective behaviors of athletes in sports teams, in adhering to specific principles of play in different performance sub-phases?

Here, it is worth alluding to the dynamical notion of an ontogenetic landscape (Thelen and Smith, 1994) to refer to the related concept of a “tactical landscape” in sport performance settings. Using the landscape metaphor, an attractor is a stable state toward which a dynamical system (such as a sports team or an individual athlete) tends to evolve (e.g., a specific collective form of organization attained in an offensive sub-phase of play) (McGarry et al., 2002; Davids et al., 2005; Boeing, 2016). Through excellent coaching, team players may be attracted toward preferred, stable states of collective organization out of many possible states (e.g., those that support team functions with principles of play).

The continuous exposition of players to instabilities associated with *phase transitions* (transitions between states of system organization, for example, in team sports, between offensive and defensive patterns) furnishes a rich landscape of tendencies in system organization for performers to exploit (Williams et al., 1999; Araújo et al., 2004; Davids et al., 2005). Cultivating a landscape of synergetic possibilities can be achieved by providing integrated fields of affordances (opportunities or invitations for action) in practice contexts [most effectively through small-sided and conditioned games (SSCG)], through which skillful goal-directed behaviors of (intelligent) team sports performers may emerge (Rietveld and Kiverstein, 2014; Araújo and Davids, 2016). This learning process can be guided by manipulating key constraints that act upon each individual, and the team, collectively (Silva, 2014). Therefore, specific key constraints like issues related with strategy and coaching (e.g., tactical principles of play) may influence the creation of functional and goal-directed synergies (entirely novel perception-action relations) among players, during competitive and learning performance environments (Araújo et al., 2014; Silva et al., 2014). By manipulating specific key task constraints during SSCGs, coaches may provide important information sources or shared affordances that enable the creation of a specific communication system, allowing self-organization to be enhanced (Silva et al., 2013). During competitive performance, team players couple to form an interpersonal synergy based on perception–action systems in a social context, underpinned by the collective perception of shared affordances (Silva et al., 2013; Araújo et al., 2014, 2015).

A “landscape of affordances” is replete with possibilities for action available in a specific performance context, related to the whole spectrum of abilities available in socio-cultural context of a sport (Bruineberg and Rietveld, 2014; Rietveld and Kiverstein, 2014). Coaches can design specific invitations for action for performers in their training sessions through manipulation of ecological constraints of practice environments. In SSCGs, local-to-global synergy formation can be enhanced between players by using constraints such as manipulating numerical relations between players, varying playing area dimensions, and scoring targets. Through use of augmented informational constraints, such as verbal instructions and feedback, coaches and teachers can stimulate specific collective patterns of behavior prescribed in principles of play related to a particular game model. In this respect, a game model founded on principles of play facilitates the utilization of specific affordances or invitations for action in performers. Such affordances (Gibson, 1966, 1979) can be perceived during important sub-phases of play and can solicit or invite specific actions or collective behaviors (Withagen et al., 2012), enabling performers to achieve intended team performance goals (e.g., exploiting open space behind or in front of an opposing defensive line or preventing scoring opportunities for opponents in a playing area by deliberately aggregating players centrally to restrict space) during competitive performance. Here, the perception of shared affordances (Silva et al., 2013) guides the formation of synergetic networks for achieving specific team goals (Araújo and Davids, 2016; Ribeiro et al., 2017).

Utilization of shared affordances is thus paramount for harnessing each player’s capacities, individually, and collectively in the team, in co-adapting to task and environmental constraints during practice and competition. Coaches should seek to promote and develop effective self-organizing tendencies at a local-to-global scale. Viewed this way, co-adaptation comprises a process that enhances, through implementing specific training methodologies, the relationship of players and teams with a competitive performance environment in specific directions. Co-adaptive moves in training enhances the fitness of individual athletes and teams in a performance environment (Silva et al., 2016), coherent with the accomplishment of intended principles of play within a game model. In this sense, the term “fitness” does not refer to conditioning but to the capacities of performers to functionally adapt to the continuous dynamical constraints of the performance task and environment in achieving performance goals.

## Conclusion and Practical Applications

We outlined that viewing athletes and sports teams as complex adaptive system entails understanding the process of synergy formation between system components during performance and practice. This process is continually shaped by exploiting inherent self-organizing tendencies in a global-to-local or local-to-global direction. Traditionally, imposition of a game model to harness tactical principles of play, by a coach, exploits global-to-local synergy formation as a major constraint on team sports performance. We highlighted the importance of coaches promoting and managing global-to-local self-organizing tendencies in teams in a flexible and adaptable manner. This approach to sport pedagogy would help develop effective local-to-global self-organizing tendencies between players, facilitating exploration of adaptive and innovative (intelligent) performance solutions coherent with the proposed principles of play circumscribed in an overarching game model. This is extremely important because for a given principle of play (e.g., exploring both right- and left-hand sides of the opposition structure to cross the ball into the penalty box), there is a multitude of possible tactical solutions [e.g., attracting the left/right defender out of his position to create available space through an individual movement (1 vs. 1), or by simply creating numerical overload (e.g., 2 vs. 1)] to be done.

Understanding the interplay between these tendencies in inherent self-organizing processes may help establish more effective athlete development programs, as well as enhancing team training and performance. Such an approach may be key in achieving the aim of developing the “intelligent performer” in sport, here defined as an athlete capable of adapting to changing performance constraints rapidly and effectively. More research is needed to theoretically and empirically scrutinize the inter-level causality evidenced by existing global-to-local and local-to-global self-organizing tendencies that shape synergy formation in team sports performance.

## Data Availability Statement

All datasets generated for this study are included in the manuscript/supplementary files.

## Author Contributions

All authors listed have made a substantial, direct and intellectual contribution to the work, and approved it for publication.

## Conflict of Interest

The authors declare that the research was conducted in the absence of any commercial or financial relationships that could be construed as a potential conflict of interest.
